# Perioperative systemic inflammation in lung cancer surgery: Just an epiphenomenon or a potential therapeutic target?

**DOI:** 10.3389/fsurg.2022.1045388

**Published:** 2022-10-17

**Authors:** Federico Tacconi

**Affiliations:** Department of Surgical Sciences, Unit of Thoracic Surgery, Tor Vergata University, Rome, Italy

**Keywords:** non-small cell lung cancer (NSCLC), VATS, systemic inflammation, minimally-invasive thoracic surgery, blood-derived inflammatory markers

**Editorial on the Research Topic** Perioperative systemic inflammation in lung cancer surgery: Just an epiphenomenon or a potential therapeutic target? By Tacconi F. (2022) Front. Surg. 9: 1045388. doi: 10.3389/fsurg.2022.1045388

The intimate interaction between inflammation and cancer is a very complex and intricate topic of general medicine. An exact description of all possible cause-effect relationships in this topic is an extremely difficult task. In fact, it is well known that, at local level, chronic inflammation may initiate tumor growth and promote progression by interacting with myriads of biological mechanisms including activation of transcription factors, promotion of angiogenesis, inhibition of anticancer adaptive immunity, and many others (1). On the other hand, tumor cells themselves may induce inflammatory changes at both local and systemic level. Tumor-induced inflammation, in turn, may enhance tumor development through autocrine and paracrine interactions, thereby generating a sort of vicious circle. Furthermore, specific inflammatory mediators may spill over systemic circulation with a potential role in metastatization (2). Based on the considerations above, much translational research has been conducted over the past 2 decades, in order to analyze the relevance of assessing systemic inflammation status in the clinical environment. The vast majority of studies showed that elevated level of inflammatory markers is usually associated with higher tumor burden and worse survival rates in various oncology conditions, including lung cancer (3). However, the practical usefulness of this association is still unclear, so that the assessment of inflammatory status has not yet implemented in the current guidelines or decisional algorithms regarding the most common solid tumors.

Furthermore, while pre-treatment systemic inflammation has been the matter of many studies, much less interest has been devoted to inflammatory events occurring in the perioperative phase. Indeed, based on the considerations above, it could be hypothesized that postoperative inflammation might act as a transient enhancement to tumor growth and metastatization. Some studies are in keeping with this basic idea. For example, Kuroda and colleagues showed that an excess postoperative increase in C-reactive protein and white-blood cell count is associated with overall and progression-free survival in 444 patients who received curative resection for stage II/III gastric cancer (4). Similar observations were shown in patients with resected non-small cell lung cancer, as well (5). The basic biological mechanisms connecting postoperative inflammation and cancer recurrence were excellently reviewed by Choi and Hwang in our Research Topic (https://www.frontiersin.org/articles/10.3389/fsurg.2022.888630/full).

A deeper comprehension about the consistency and the magnitude of the relationship between postoperative inflammation and cancer progression might help develop strategies aimed at optimizing outcomes, thanks to the implementation of mitigating strategies. In this regard, a mulicentric randomized controlled trial is ongoing in Japan, aimed at investigating the usefulness of flurbiprofen axetil for preventing recurrences after lung cancer resection (Japan Registry of Clinical Trials: 031190167) (6). Other strategies may be the use of different surgical and anesthetic techniques associated with an attenuated inflammatory perturbation.

When we proposed this Research Topic, our scope was to shed more light on the questions above. The contributors went well beyond the expectations and provided us with a number of surprising and innovative observations that increased remarkably our degree of acquaintance with this theme.

Wang and colleagues provided us with their results of a massive analysis of 995 patients https://www.frontiersin.org/articles/10.3389/fsurg.2022.830642/full). They showed that, amongst a series of clinical and lab indicators of systemic inflammation, increased basophil-to-neutrophil ratio and low albumin-to-globulin ratio were the most reliable predictors of poorer survival, with a fair discriminant ability. But even more importantly, this study should be credited for the use for its refined statistical approach. In this regard, we would like to seize the opportunity to welcome the use of advanced computational algorithm to optimize the interpretation of results in a so complex topic, as the potential for countless spurious associations may easily lead to misleading conclusions.

Furak and colleagues offered a panoramic review of the impact of systemic inflammation and immune changes after lung cancer surgery, with a special view to possible role of mitigating strategies including minimally-invasive surgical approaches and/or the use of spontaneous ventilation (https://www.frontiersin.org/articles/10.3389/fsurg.2022.883322/full#B58). In this regard, interesting observations also come from the Yokohama's group, where Watanabe and his coworkers found worse survival rates in patients who received volatile anesthetics, as well as a protective effects of intravenous non-steroidal anti-inflammatory drugs (https://www.frontiersin.org/articles/10.3389/fsurg.2022.886241/full).

With respect to anti-inflammatory strategies, much relevance should be given to the study of Yang and colleagues from the Tonji Medical College of Wuhan, China. The authors showed a detrimental effect of multiple glucocorticoid administration on postoperative cell-mediated immunity (https://www.frontiersin.org/articles/10.3389/fsurg.2022.859984/full). Based on these results, a caveat on the use of glucocorticoids as inflammation-targeting strategy should be raised, even though they have been sporadically associated to improved survival rates in other surgical oncology settings (7). Finally, other than pure oncological implication, taking systemic inflammation status into account might help predict hospital re-admission with a potential for tailored early follow-up, as shown by Luo and colleagues (https://www.frontiersin.org/articles/10.3389/fsurg.2022.893555/full).

We sincerely thank all the authors for their outstanding contributions. Our hope is that Frontiers' readership may find our Research Topic useful and of inspirational for further and more focused investigations. Targeting postoperative inflammation might be one of the possible keys of an optimized patient management in the era of precision medicine, and we are proud of having given a little help in this process.
